# The medical cyborg concept

**DOI:** 10.14806/ej.27.0.1005

**Published:** 2022-04-21

**Authors:** Eleni Papakonstantinou, Thanasis Mitsis, Konstantina Dragoumani, Flora Bacopoulou, Vasilis Megalooikonomou, George P. Chrousos, Dimitrios Vlachakis

**Affiliations:** 1 Laboratory of Genetics, Department of Biotechnology, School of Applied Biology and Biotechnology, Agricultural University of Athens, Athens, Greece; 2 University Research Institute of Maternal and Child Health & Precision Medicine, and UNESCO Chair on Adolescent Health Care, National and Kapodistrian University of Athens, “Aghia Sophia” Children’s Hospital, Athens, Greece; 3 Computer Engineering and Informatics Department, School of Engineering, University of Patras, Patras, Greece; 4 Division of Endocrinology and Metabolism, Center of Clinical, Experimental Surgery and Translational Research, Biomedical Research Foundation of the Academy of Athens, Athens, Greece

## Abstract

Medical technology has made significant advances in the 21^st^ century and, at present, medicine makes use of information technology, telecommunications, and state-of-the-art engineering to provide the best possible healthcare services. Electronic sensors provide health practitioners with the ability to constantly monitor their patients’ health, to streamlines a number of medical processes, and to increase patients’ access to health services. Mobile phones also empower patients and play a major role in their health’s monitoring. The use of cybernetics technology can now help patients overcome even serious disabilities, enabling many disabled patients to live their lives similarly to their non-disabled fellow men through the use of artificial organs and implants. All these advances have paved the way for a more personalized type of healthcare that provides individualized solutions to each patient. Once a number of hurdles are overcome, medical technology will bring forth a new era of more precise and enabling medicine.

## Introduction

The term medical technology refers to the application of scientific knowledge and skills in the form of devices, medicine, vaccines, procedures, and systems made to solve a health problem and improve patients’ quality of life (Ten Haken et al., 2018). The technological developments observed in the 21st century have led to tremendous changes in healthcare (Thimbleby, 2013). What could be thought of as science fiction a couple of decades prior is, now, everyday medical practice. From the use of robots (Hockstein et al., 2007) to tailoring therapy to each patient’s needs (Mathur and Sutton, 2017), modern medicine could be considered an advanced technological profession (Mesko, 2018). This notion is further reinforced by the introduction of modern engineering, information technology, and wireless communications in everyday medical practice.

The above technological developments have led to the emergence of novel medical fields and concepts. This review summarizes a number of ways technology has influenced current medicine and displays a number of promising future prospects.

## eHealth

Electronic health (eHealth) refers to the medical field, where information and communications technologies are used in health-related services and processes. eHealth includes a variety of applications, such as electronic health records, electronic medication overviews, medical data collection, and telemedicine services (Wernhart et al., 2019).

### Electronic health records

Electronic health records (EHRs) refer to the digital forms of patient care records that include information, such as personal contact information, medical history, medication orders, diagnostic and laboratory test results, allergies, and treatment plans (Kruse et al, 2018; Ratwani, 2017). EHRs bring multiple benefits to medical practitioners. First, EHRs allow the use of computerized clinical decision support systems (CDSSs) (Menachemi and Collum, 2011). Such systems consist of software designed to aid in clinical decision-making, where an individual patient’s characteristics are matched to a clinical knowledge database and patient-specific assessments or recommendations are given to the clinician for the betterment of her/his decision (Sutton et al., 2020). Another benefit of EHRs is the incorporation of computerized physician order entry (CPOE) (Menachemi and Collum, 2011). CPOE systems are computer applications that offer clinicians the ability to enter electronic orders for medications, laboratory tests, imaging examinations, medical procedures, and referrals (Amiri et al., 2018). The digitization of these processes reduces medication errors potentially caused by clinicians’ poor penmanship and makes ordering more efficient, because nursing and pharmacy staff do not need to pursue clarifications or retrieve missing information from illegible or incomplete orders (Menachemi and Collum, 2011).

EHRs allow for the secure exchange of medical information among different health organizations and providers, promoting synergy and cooperation. This exchange’s results are ideally the improved speed, accuracy, cost, and safety of medical decisions (Devine et al., 2017). Last, information from EHRs can be used for secondary research purposes also. A prime example is that data in EHRs can be mined to identify previously unknown drug interactions or adverse events, which are essential research topics in pharmacology (Carroll et al., 2015). Although the use of EHRs seems to be constantly increasing among healthcare providers and organizations, a number of obstacles need to be overcome. First, although legal protections have been implemented, EHRs are prone to breaches that may harm patient privacy (Shenoy and Appel, 2017). Second, although EHRs are generally thought to decrease the risk of medical errors, simple actions made possible through computerized record-keeping, like copy and pasting, may cause repeated typing errors that can potentially lead to a medical error (Palabindala et al., 2016). Last, the cost of implementation and maintenance is quite high, which disincentivizes hospitals and healthcare providers from using EHRs (Palabindala et al., 2016).

### Wearables

A significant method of medical data collection, analysis, and storing is through wearables that accumulate real-time patient data. Wearables provide helpful information to prevent, diagnose, monitor, and manage chronic diseases and conditions (Uddin and Syed-Abdul, 2020). These devices include wristbands, smartwatches, wearable sensors, and mobile hub medical devices that collect data such as heart rate, skin temperature, galvanic skin response, skin temperature, peripheral capillary oxygen saturation, plus geolocation information and ambient environmental variables (Heikenfeld et al., 2018; Witt et al., 2019). The ability to provide real-time data is one of the most important assets presented by wearables. Most diagnostic tools provide information that is ‘a snapshot in time’ while wearables allow continuous monitoring of physiological and biochemical information under natural physiological conditions and in any environment (Aliverti, 2017).

Naturally, the information received in the ‘snapshot’ period may not be representative of a patient’s health status, while monitoring a week’s worth, or longer, of data can improve the analysis of the patient’s health and help elucidate the progress of an existing disorder. The constant monitoring of an individual’s current health condition by clinicians is vital in patients with chronic diseases, such as Alzheimer or Parkinson disease, or patients who are in a critical condition (Hasan et al., 2019). In these cases, information received by wearables can be used to adjust medication dose, manage possible adverse events, and check patient’s adherence to medical advice (Izmailova et al., 2018).

In addition, wearables can be used by sports physicians. Wearables can provide information on an individual athlete’s movement and physical activity, allowing sports physicians to design more efficient training programs for optimal performance (Li et al., 2016). A prime example is providing real-time feedback to swimmers and runners in an effort to better their technique (Adesida et al., 2019). The constant monitoring of an athlete’s workload and biological parameters can help minimize the potential of injury or mitigate the effects of any existing one (Seshadri et al., 2019).

Although wearables are quite promising devices when it comes to their application in eHealth, they do have a number of disadvantages. A recurring theme in the current review regarding the shortcomings of modern medical technology is privacy concerns. Wearables accumulate a large number of personal health data, where potential breaches can lead to the exploitation of patients’ medical information (Cilliers, 2020). Moreover, wearable devices may lead to unintentional behavioral changes, like patients becoming overly anxious about their health and display an “addiction” to the wearable device (Schukat et al., 2016).

### Telemedicine

The term telemedicine refers to the delivery of healthcare services at a distance through the use of electronic technology (Serper and Volk, 2018). Telemedicine includes various practices such as over-the-phone consultation, medical advice via video-calls or e-mails, access to and sharing of medical data, and telesurgery (Zhang and Zhang, 2016). Numerous medical branches make use of telemedicine, such as radiology, pathology, dermatology, and psychology (Kruse et al., 2017). Medical practitioners provide three distinct types of telemedicine. The first one is synchronous telemedicine, where patient and provider have a live interaction. The second one is asynchronous telemedicine, where a patient or physician stores medical history, images, and pathology reports and then forwards them to a specialist for diagnostic and treatment expertise. Last, there is remote patient monitoring, where a physician continuously monitors a patient’s health through direct video monitoring or reviewing continuous data received remotely (Mechanic et al., 2020). Interest in telemedicine is rising since it extends the services of healthcare providers to remote areas and increases the availability of experts in specific medical fields (Kruse et al., 2017).

Telemedicine showcases a number of disadvantages such as privacy concerns, but the most important is the occasional data transmission delay. In this case, the lack of dedicated and reliable networking infrastructure may lead to delays in information exchange, potentially postponing critical diagnoses and interventions (Gang et al., 2017).

As mentioned above, an important application of telemedicine is telesurgery, which utilizes wireless networking and state-of-the-art robotics to allow surgeons to operate on patients who are distantly located (Choi et al., 2018). Telesurgery is really important for patients who cannot afford to move out of their residence for various reasons, including risky travel, economic burden, and health reasons. Despite its’ promising nature, telesurgery still remains at a halt due to lack of training programs, equipment expense, and legal issues among countries (Choi et al., 2018). Thus, currently, the main method encompassing eHealth into surgical procedures is telemonitoring, where an expert surgeon guides another surgeon in a different geographical location by watching a real-time feed of the operation (Hung et al., 2018).

## mHealth

The advent of smartphones and tablets, mobile personal devices with computing abilities and access to the internet, seem to influence every aspect of modern life (Panova and Carbonell, 2018). These devices use microprocessors that provide computing power similar to desktop personal computers but on a smaller size and with a lower power budget (Furber, 2017). This increase in computing power and mobile connectivity has led to the emergence of a new technological field called mobile health (mHealth) (Steinhubl et al., 2015). Mobile health refers to the use of mobile devices and applications (apps) to deliver healthcare services (Wilson, 2018b). These devices and applications are used both by healthcare providers and patients and have an essential role in healthcare democratization. Regarding their role in healthcare providers’ work, mHealth applications allow mobile devices to function as wearable sensors or health communication hubs, becoming an integral part of the eHealth field.

Furthermore, it is expected that through modern advancements in microfluidics and microelectronics, mHealth devices could potentially act as mobile labs with diagnostic capabilities (Steinhubl et al., 2015). Regarding these devices and apps’ effect on patient life, the use of mHealth apps seems to have an empowering role and can help these patients manage their health. These applications are cost-effective, help patients track their health status, increase patient adherence to medical advice, and promote healthier lifestyle decisions (Mahmood et al., 2019). These characteristics of mHealth make this technology very intriguing, however, there are several obstacles that deter mobile health from reaching its’ full potential. Apart from the disadvantages present in wearables technology, which are also characteristics of mobile devices, health apps add new complications to the application of mHealth. These apps are currently not adequately regulated, with many of them overpromising on their healthcare potential or even being outright deceitful, thus endangering an individual’s health (Steinhubl et al., 2015).

## The cyborg concept

The modern advancements of medical technology are more evident than ever when nowadays, the term ‘cyborg’ does not feel like a far-fetched science fiction concept, but a word describing everyday people (Quigley and Ayihongbe, 2018). Cyborg, short for cybernetic organism, refers to an organism that includes both biological and electronic parts (Li and Zhang, 2016). Current bionic technologies like bionic hands, leg prostheses, exoskeletons, retina-implants, and cochlear-implants have helped numerous individuals with physical disabilities (Meyer and Asbrock, 2018). People who were once perceived as ‘good intentioned but lacking in physical abilities’ are now thought to be equally competent -or at times- more competent than non-disabled individuals (Meyer and Asbrock, 2018).

Modern-day bionic hands allow motor control of prostheses, while rigorous research is being conducted on adding the ability of intricate sensory feedback (Bumbaširević et al., 2020). These so-called myoelectric prostheses use embedded electromyography (EMG) electrodes that record the muscle’s electrical activity and use it to control the prosthetic limb (Aman et al., 2019). Although currently commercially available myoelectric prostheses do not offer any intentional sensory feedback, experimental approaches that use neural interfaces to stimulate peripheral nerves have been shown to elicit sensations such as pressure and pain (Aman et al., 2019). Moreover, advancements in embedded systems technology appear to be quite promising regarding the betterment of control and feedback of such prostheses (Mastinu et al., 2017). On the other hand, lower-limb prostheses (LLPs), like a prosthetic foot, use mechanical joint axes, compressive foams, and bumpers (Stevens et al., 2018). Lower limb extremities are less complicated than the upper limbs ones, and thus patients receiving LLPs can walk, dance, or participate in sports on a level similar to that of non-disabled individuals (Bumbaširević et al., 2020).

Exoskeletons, *i.e.*, wearable robotic units controlled by computer boards that power an intricate mechanical system to restore locomotion, are mainly used for rehabilitation purposes in medical centers or home use (Gorgey, 2018). Research has shown that the use of exoskeletons may have beneficial effects on gait function and walking independence in a mixed population of neurological disorders (Palermo et al., 2017). Electronic retinal implants are a step towards artificial vision. Artificial vision attempts to enable some blind people to see through the electrical stimulation of the retina (Mills et al., 2017). Retinal implants have shown a number of promising results, such as partial visual restoration and better performance in everyday tasks (Bloch et al., 2019).

Cochlear implants are used to treat children and adults with severe to profound sensorineural hearing loss (Deep et al., 2019). These implants transduce acoustic energy to an electrical signal that they later use to stimulate the auditory nerve’s surviving spiral ganglion cells (Deep et al., 2019). Cochlear implants are some of the most successful prostheses and have helped a tremendous number of people around the world (Wilson, 2018a). Lastly, implantable biosensors provide accurate real-time health assessment of a patient, with a prime example being implantable glucose monitors that can assess glycemia in real-time in diabetes (Waldman and Terzic, 2011).

The next step for cybernetics and medicine would be the construction of fully artificial internal organs. Several events have taken place in the last couple of decades towards the accomplishment of this goal. 3D printing, a relatively new technology that generates three-dimensional constructs from digital information, is an essential part of the scientific effort to produce artificial internal organs (Aimar et al., 2019). 3D-bioprinting particularly allows printing different cell types, biomolecules, and biomaterials simultaneously (Pati et al., 2016). Potential fully bio-printed organs could save lives by reducing the waiting list of patients in need of organ transplantation (Aimar et al., 2019). Currently, synthetic organ devices like artificial hearts, such as the SynCadia Total Artificial Heart, are used to support patients’ circulatory system, thus allowing sufficient time to find appropriate live heart transplants (Chung et al., 2020).

In any case, bionic implants still showcase a number of disadvantages that should be taken into consideration. Just like regular transplantation, there is a possibility of infection (Bumbaširević et al., 2020; Lewis et al., 2016). This possibility makes the already highly intricate attachment of a bionic part even more difficult and demands constant monitoring after the procedure. Another potential problem is the interference caused by electronic devices. Multiple studies implicate novel devices like wireless charging systems that can generate electromagnetic fields in the intermediate frequency which could interfere with cardiac electronic implants (Driessen et al., 2019). Last, many patients may themselves reject such technology for reasons such as high cost, durability, appearance, and the already mentioned lack of intrinsic feedback functions (Godfrey et al., 2018).

## Towards personalized medicine?

The so-called holy grail of medicine has always been to provide ‘the right treatment to the right patient at the right time’ (Kravitz, 2014). Recent technological advancements have set the basis for the development of more personalized medicine, one which uses both modern nucleic acid sequencing techniques and some of the aforementioned monitoring and implantation methods to individualize treatment (Goetz and Schork, 2018).

The emergence of next-generation sequencing techniques has allowed the quick and cheap sequencing of a complete human genome (Garrido-Cardenas et al., 2017). By sequencing a patient’s whole genome, a genomic portfolio could be constructed that showcases their possible predispositions and vulnerabilities to certain diseases (Mathur and Sutton, 2017). This information could later be included in their EHR, giving a clinician a more precise image of the patient’s health (Williams et al., 2019). A patient may then be monitored for specific biomarkers or behaviors that are characteristic of the pathophysiology to which they are susceptible. The monitoring could be undertaken by wearables, mobile health applications, implants, or telemedicine sessions. The above procedures may allow a clinician to propose an intervention completely aimed that specific patient for optimal outcome.

## Conclusions

**Figure 1.**
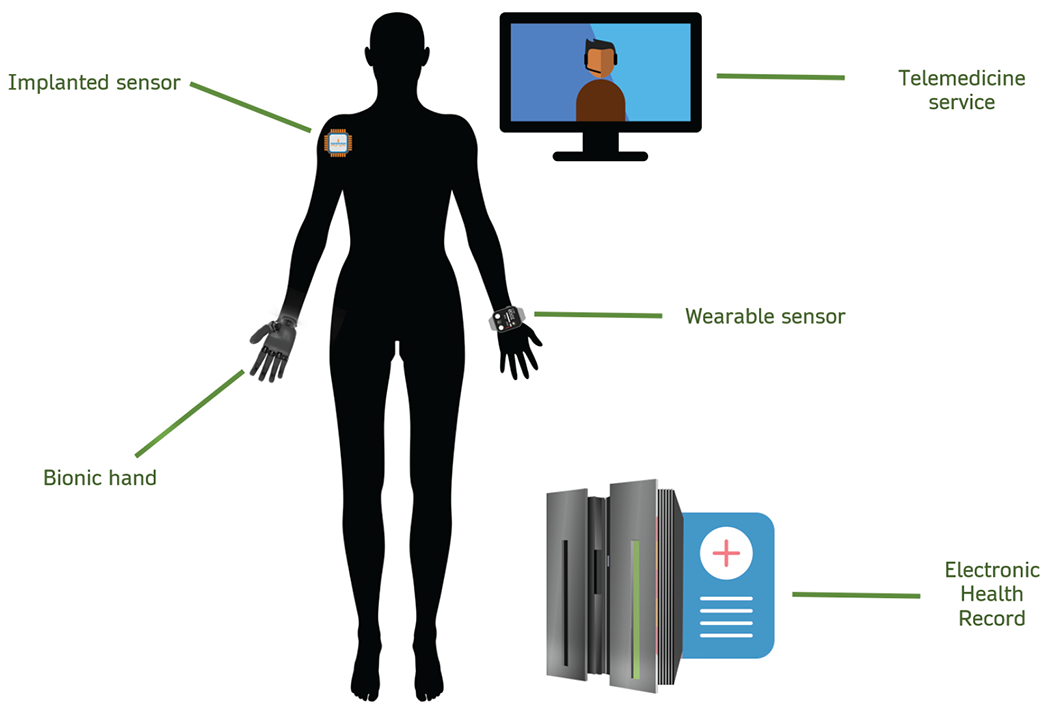
Modern healthcare as influenced by recent advancements in medical technology. The use of bionic limbs could potentially overcome serious disabilities. Implanted sensors can provide real-time information regarding complex biomarkers like glucose levels, while wearables can provide information on everyday fluctuations of health markers, such as body temperature and heart rate. This information could then be included in an extensive HER. Telemedicine services could then provide a patient with the appropriate advice based on the aforementioned information.

Medical technology has evolved by leaps and bounds in the 21st century. Information technology, telecommunications, and the ever-advancing power of computer processors have granted clinicians the ability to monitor an individual’s health for great periods of time, or even continuously, as opposed to the ‘snapshot’ visits at a physician’s office. Moreover, the inclusion of electronic health records and genomic profiles in modern hospitals allows health practitioners to tailor their therapeutic approach to each individual patient. The use of bionic technology can now overcome even life-altering disabilities. These advancements have led to the emergence of new and more precise healthcare services (Figure 1).

Just like all technological advancements, a number of hurdles should be overcome. The most important one is privacy. A patient’s terminal disease, sexually transmitted disease, or even genetic profile could be used as a means of extortion by criminals. The storage and sharing of sensitive medical information should be a high priority in the coming years. Another problem is the cost of research. Medical technology is a complicated high-risk field that requires large investments. Moreover, the application of modern technology also has a high cost, both by health organizations and by customers. On the other hand, expensive technology is a bargain if it can improve quality of life, preserve economic productivity, and prevent the high cost of disability. Last, medical technology should aim to help as many people as possible, thus, attributes like ease of use should be taken into account in order to provide the best possible service.

## Key Points

- Cybernetics technology will enable the holistic medical, preventive and personalized perspective of health care.
- Electronic health records (EHRs) are the digital form of patient care records that set the ground for a digitalized decision support system.
- Bionic technologies like bionic hands, leg prostheses, exoskeletons, retina-implants, and cochlear-implants have helped numerous individuals with physical disabilities.
- The concepts of eHealth and mHealth inclusion in the healthcare delivery system pave the way for a tailored therapeutic approach.
